# EVAC: Evacuation of Vulnerable and Critical Pediatric Patients for Nurses

**DOI:** 10.7759/cureus.8302

**Published:** 2020-05-26

**Authors:** Anita Thomas, Megan M Gray, Brian Burns, Rachel Umoren

**Affiliations:** 1 Pediatrics, Seattle Children's Hospital, Seattle, USA; 2 Neonatology, Seattle Children's Hospital, Seattle, USA; 3 Pediatric Emergency Medicine, Seattle Children's Hospital, Seattle, USA; 4 Pediatrics, University of Washington School of Medicine, Seattle, USA

**Keywords:** evacuation, disaster, neonatal, pediatrics, nursing education, simulation, emergency preparedness

## Abstract

Disasters such as earthquakes can interrupt healthcare delivery by forcing the evacuation of intensive care patients. Critically ill neonates are particularly vulnerable due to their complexity and thus can be difficult to safely and efficiently evacuate in a disaster. In general, most education surrounding this is based on lectures. This technical report describes the creation and use of a simulation-based curriculum focusing on the evacuation of a critically ill, septic neonate by a single nurse participant in the setting of an earthquake. This simulation provides learners the experience of expediently assessing safety in the setting of a disaster and prioritizing equipment when evacuating a critically ill neonate, which may provide a more realistic training environment than traditional lectures.

## Introduction

Unexpected catastrophic events such as earthquakes interrupt essential healthcare delivery via structural damage, flooding, and staff and supply shortages that force the evacuation of intensive care patients [1-2]. Critically ill neonates are some of the most vulnerable patients in disasters, given their often complex medical management involving multiple lines, airway maintenance, medications, thermoregulation, etc. [3]. The purpose of this curriculum is simulation education of nurse-led evacuation of a critically ill neonate in the setting of a disaster. Effective evacuation of this simulated septic neonate requires knowledge of safe and efficient patient packaging and transport.

The primary goal of pediatric patient evacuation is to safely and effectively transport the patient. Given the critical nature of a septic neonate, it is important to ensure that the necessary equipment is transported to maintain the patient. Septic-intubated neonates who are hemodynamically unstable require antibiotics, intravenous fluids, vasopressors, and airway equipment for adequate therapy [3-4]. Along with critical care equipment, other vital materials for safe transport in a disaster are required such as patient identification. 

## Technical report

Methods

The design of this simulation incorporated adult learning principles including allowing for active learning through participation and immediate feedback. This simulation allows participants to individually perform an environmental assessment in the immediate aftermath of an earthquake, practice communication via a repeat back, and demonstrate appropriate patient preparation for evacuation. At the conclusion of the scenario, facilitators crosscheck a list of items and review actions taken with medications, infusions, tubes, drains, and patient information.

This simulation case was developed to help nurses systematically prepare to evacuate a critically ill neonate as part of the pediatric nursing disaster curriculum. To differentiate this simulation exercise from standard hospital disaster curriculum, the authors entitled the case Evacuation of Vulnerable and Critical Pediatric Patients (EVAC). Through participation in this simulation, learners have the opportunity to practice communication with their charge nurse, assessment of the environment, themselves, and the patient in the event of an earthquake, as well as practice preparing the patient for evacuation by appropriately managing bedside patient equipment necessary for patient survival. Though it was originally developed for a target audience of pediatric nurses in the neonatal intensive care unit (NICU) or pediatric emergency department (PED) setting, it may be modified for other healthcare personnel that may be charged with evacuating a critically ill neonate, such as pediatric intensive care unit nurses or general emergency medicine nurses. Ideally, nurse participants should have prerequisite knowledge of general patient assessment, common healthcare communication tools, and understanding of general patient transport. Depending upon your targeted learners, it might be helpful to review these before the simulation [3-6].

**Figure 1:**
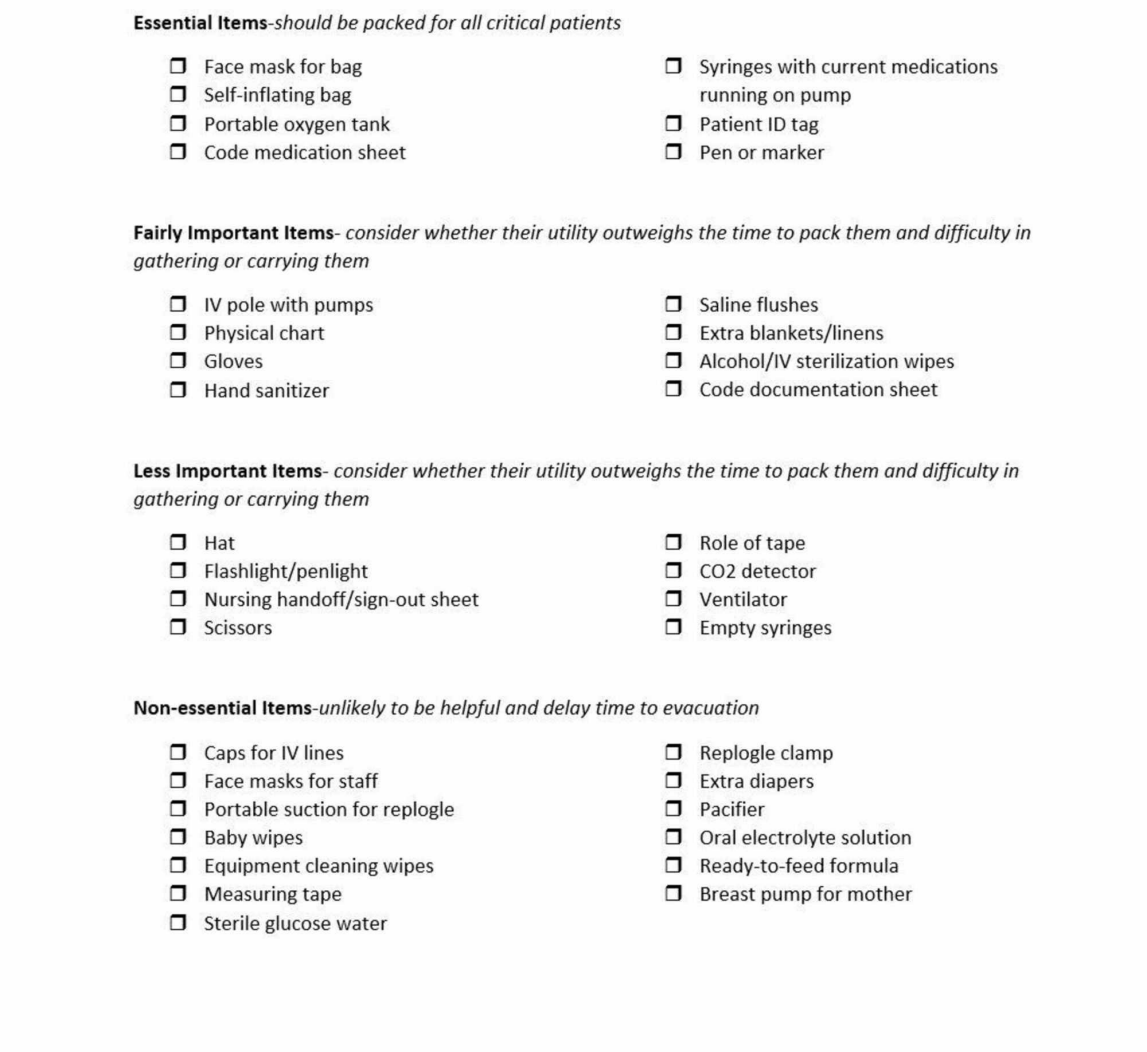
Critical items checklist Facilitators may use this checklist to track which items are being collected. This may be useful to share with participants as a visual tool as well.

A checklist (Figure 1) was created to help score pediatric hospital equipment needed to evacuate a critically ill neonate via a modified Delphi method by surveying experienced charge nurses from the neonatal intensive care unit and the pediatric emergency department, with a necessity ranking ranging from most critical equipment to not important equipment [7].

After the scenario is completed, facilitators may utilize the debriefing materials in Appendix A as a guide. Feedback on the case may be obtained verbally and via the evaluation form provided in Appendix B. Appendix C is optional and includes a slides-based didactic put together by the authors as supplemental to facilitator-led teaching and debriefing. We received feedback that it may be helpful for participants to have a checklist during the simulation or debrief provided and so Appendix D: EVAC Simulation Checklist Card was created for participants (may be printed front and back and downsized to identification (ID) card sized). Lastly, pediatric disaster communication terminology is provided in Appendix E for the instructor in preparation for the simulation case and to utilize in prebriefing and/or debriefing.

Primary Educational Objectives

By the end of this activity, learners will be able to:

1. Evaluate the post-earthquake environment by checking themselves, identifying hazards, and checking on their patients.

2. Efficiently organize appropriate materials for safe patient transport and evacuation.

3. Demonstrate effective communication while assessing a patient for evacuation during a disaster using standard communication tools such as SBAR (situation, background, assessment, recommendations) and closed-loop communication. 

Case Summary

Brandon is a 15-day-old male who presented with fever and was found to have sepsis. He is intubated, receiving antibiotics, vasopressors, and intravenous (IV) fluids. The patient is on full monitors and has on a diaper and hospital gown. After receiving sign-out on this patient, an earthquake occurs and the nurse participant is expected to prepare the patient for evacuation.

Anticipated interventions include assessment of the environment/self/patient, clear communication, and collecting appropriate items for patient evacuation. The patient’s vital signs remain static throughout the case, and the participant has five minutes to complete the evacuation.

Learner Critical Actions

1. Assessing self, environment, and patient

2. Utilizing clear communication with the charge nurse

3. Collecting at least all essential items on a checklist

4. Preparing to evacuation in less than five minutes

5. Disconnecting the patient from the ventilator and utilize handbag masking

6. If utilizing an embedded participant parent, clear and open communication to the parent

Personnel

Instructor: Bedside Nurse who transitions into the role of Charge Nurse

If other instructors are available, one may play the bedside nurse and the other may play the charge nurse.

If available, an embedded participant may play the patient’s parent, so that communication with the parent may be assessed.

The simulation scenario is designed as a curriculum for a sole learner with a target audience of nurses that care for critically ill neonates, such as pediatric emergency medicine (PEM) or NICU nurses. There are no prerequisites for nurses prior to participating in this case. Depending on the nurse’s background, facilitators may provide optional background material on disasters and patient evacuation (Appendix C), as well as common healthcare communication tools (Appendix E). Educational materials may be provided either before or after the scenario. The facilitator plays the role of the charge nurse giving patient sign-out as well as informs the participant of the earthquake and provides instructions regarding evacuation. In our curriculum, facilitators were physicians; however, the scenario may be led by nurses in educational roles, such as unit nursing educators, charge nurses or nursing managers, thus improving the realism of the scenario. 

Learners

Bedside Nurses

Learner Preparation

1. Introduction to pediatric disaster training

· Emergency Nurses Association (ENA). ENPC: Emergency nursing pediatric course: Provider manual. 4th ed. Des Plaines, IL: Emergency Nurses Association; 2012.

2. General knowledge about pediatric evacuation and earthquake preparedness

· Monteiro S, Shannon M, Sandora TJ, Chung S. Pediatric Aspects of Hospital Preparedness. Clinical Pediatric Emergency Medicine. 10(3) 216-28; 2009.

· FEMA. Earthquake Preparedness: What every child care provider needs to know. 240; 2006.

· EVAC Simulation Slide-based Didactic Video (Appendix C)

Environmental Set-Up and Implementation

The setting is PED resuscitation room or NICU, and the simulation can be conducted in situ or in a simulation lab using either a high-fidelity or low-fidelity manikin. We used a low-fidelity manikin.

Environmental Preparation

Plan to arrive a minimum of one hour prior to the participant and set up the room in "disaster mode" with an infant manikin (high fidelity or low fidelity depending on institutional resources) and the following required items. Rooms can be turned over within five minutes after each participant has successfully evacuated. Please see Table 1 below for a checklist for set up.

**Table 1:** Room setup in post-earthquake mode NICU, neonatal intensive care unit; ED, emergency department; IV, intravenous; CO_2_,_ _carbon dioxide; BP, blood pressure; ECG, electrocardiogram; NS, normal saline

Room setup	On warmer (NICU) Or bed (ED)	Next to warmer (NICU) Or bed (ED)	On cart/tray away from bedside	By sink
Overturned trash	Sim baby with one to two peripheral IVs	IV pole with five infusion pumps	Nursing chart	Face masks for staff
Cabinets open	Peripheral arterial line (NICU)	D10W bag	Nursing handoff sheet	Hand sanitizer
Drawers open	Arterial line tubing/fluid (NICU)	Labels for saline, D10 maintenance, heparin, antibiotics, dopamine	Code medication sheet	Cleaning wipes
Phone off hook	IV extension tubing	Three 10mL syringes	Blank code sheet	Blankets in cabinet above sink
Chair overturned	IV holder	NS bag	Pen or marker	
Black cracks on walls (may use black construction paper taped to wall)	Baby gown	Portable Oxygen tank	Flashlight/penlight	
Fake ceiling tile on floor (may use cardboard)	Pacifier	Cardiac monitor	Extra labels	
Blood bag with red food coloring spilled on floor	Ambubag	Conventional ventilator	Measuring tape	
Hazardous waste overturned	Ventilator	IV tubing for fluids	Alcohol swabs	
Water faucet leaking	CO_2_ detector		Scissors	
	Ambubag Face Mask, large		Calculator	
	Ambubag Face Mask, small		Tape	
	Replogle tube		Caps (blue)	
	Blue replogle clamp		Saline Flushes (two 3mL syringes) Diapers	
	Patient identification		Baby wipes	
	Hat		Oral rehydration fluid	
	Suction		Glucose water	
	Pulse oximeter		Formula	
	ECG leads		Gloves	
	BP cuff on leg		Breast pump kit	
	Thermometer		Extra blanket/Linens	

With a low fidelity manikin, vital signs may be provided verbally or via a simulator application for a phone or tablet. Physical exam findings can be described concurrently with the learner’s examination of the manikin. The patient is in the process of treatment and the nurse participant is getting a patient sign out. He is a critically ill septic neonate that is endotracheally intubated and thus sedated and minimally responsive. He is receiving antibiotics, IV fluids, and a dopamine infusion (Table 2).

**Table 2:** Initial patient presentation HR, heart rate; PEEP, positive end-expiratory pressure; NICU, neonatal intensive care unit; ED, emergency department

Initial Presentation			
Initial vital signs	HR 160 Oxygen saturation (SpO_2_) 100% Blood Pressure (BP) 70/50 Respiratory Rate (RR) 30 Temperature (T) 37.5 degrees Celsius Ventilator settings: Assist Control (AC) 6 mL/kg; PEEP 6, RR 44, Fraction of Inspired Oxygen (FiO_2_) 0.45		
Overall Appearance What do learners see when they first enter the room?	Brandon was brought to the hospital by his parents for a fever. He has been diagnosed with neonatal sepsis and has been sedated, intubated, has intravenous (IV) access and is receiving IV fluids, antibiotics, and a blood pressor medication. He is awaiting transfer to the NICU (ED setting) or has just settled into his admission bed in the NICU (NICU setting). Upon entering the room, the learner sees it in disarray from an earthquake with various items turned over or on the floor, cracks in the walls, etc. as described above. The patient is unharmed from the earthquake. The only people in the room initially are the facilitator and the participant. The facilitator will play the role of the charge nurse, go over instructions, await a repeat-back from the learner, and then allow the learner five minutes to prepare the patient for evacuation. The facilitator should leave the room during the preparation for the evacuation process to emulate realism.		
Actors and roles in the room at case start Who is present at the beginning and what is their role? Who may play them?	Bedside Nurse: Learner Instructor: Bedside Nurse and then Charge Nurse. If other instructors are available, one may play the bedside nurse and the other may perform the role of charge nurse. If available, an embedded participant may play the patient’s parent, so that communication with the parent may be assessed.		
HPI Please specify what info here and below must be asked vs. what is volunteered by patient or other participants	Brandon is a 15-day-old septic male infant who is intubated, getting antibiotics, intravenous fluids, and pressors. He is beginning to stabilize after these interventions, however, will require NICU level care for continued treatment. Start prebriefing just outside the room with the following instructions: “For this simulation, you will be the bedside nurse of a critically ill patient who requires the evacuation from the hospital during a disaster. Your goal is to quickly and safely prepare your patient for evacuation by packing equipment and supplies you feel would be necessary for the situation. I will give you sign out on your patient. After you receive the sign-out, you may enter the patient room. Do you have any questions before we begin?” Provide the following information: “Your patient Brandon is a 2.5 kilogram, 15-day old former 38-week-old male. He presented to the ED with fever and is being treated for sepsis and shock. He was intubated and placed on a conventional ventilator for apnea. He is hemodynamically unstable and has received 40mL/kg of normal saline (NS) boluses with another 20mL/kg NS bolus running now. He is on a dopamine infusion with stable blood pressure. I just started his first dose of antibiotics. He is currently on maintenance IV fluids with D10W for some initial hypoglycemia which is now stable. He has received sedation and is minimally responsive. “ For ED simulation: “Brandon is currently awaiting a bed in the NICU. His mother has just stepped out of the unit to make a phone call.” For NICU simulation: “Brandon is just getting settled into the NICU after admission from the ED. His mother has just stepped out of the unit to make a phone call.” If asked about events leading up to the presentation (SAMPLE history): Signs/symptoms: Brought in by parents for a fever to 101 deg F at home without other symptoms Allergies-None Medications-None Past Medical History-Full term, no complications, received Vitamin K shot and Hepatitis B immunization shortly after birth. No hospitalizations or surgeries. Last meal: Breast milk about one hour prior to presentation. Events preceding: Mother was feeding the patient and noted that he felt warm and so took a rectal temperature and it was noted to be elevated. If asked about emergency department course: The patient presented febrile to 40 degrees Celsius in the ED and was noted to be mottled with hypotension, tachycardic, and had apneic periods. He received 40 mL/kg of normal saline (NS), antibiotics, and was intubated for apnea with rapid sequence intubation and continues to be sedated. Dopamine was started to maintain appropriate blood pressures and maintenance intravenous fluids with D10W were initiated for initial hypoglycemia of 41, now normalized at 100. For access, the patient has two peripheral intravenous lines (ED) or one peripheral intravenous line and one arterial line (NICU). He is running maintenance intravenous fluids and a 20 mL/kg NS bolus.		
Past Medical/Surgical History	Medications	Allergies	Family History
Born at 38 weeks gestational age via uncomplicated vaginal delivery. The pregnancy was uncomplicated. The patient had no difficulties at birth and was discharged from the hospital on day of life 2. No past surgical history.	None	No known drug allergies	None
Physical Examination			
General	Unresponsive with sedation		
HEENT	Patent, intubated airway, attached to ventilator, oral gastric tube in place.		
Neck	Supple		
Lungs	Respirations as set by ventilator, clear breath sounds bilaterally. No stridor, crackles, or coarse breath sounds.		
Cardiovascular	Regular rate and rhythm, 2+ distal and central pulses, capillary refill 2-3 seconds, warm skin		
Abdomen	Soft, non-tender, non-distended, umbilicus appears clean, dry and without redness		
Neurological	Pupils are 3-->2mm reactive bilaterally. The patient is unresponsive to painful stimuli secondary to sedation. Low tone secondary to sedation. Glasgow Coma Scale of 3 (if asked)		
Skin	No rash, bruises, or mottling. Two peripheral intravenous lines in place (ED), One peripheral venous line and one arterial line (NICU)		
GU	Normal GU exam		
Psychiatric	Unable to assess		

The critically ill neonate should be set up with a low fidelity infant mannikin with initial vital signs and appropriate bedside equipment. An IV pole with medications labeled should be attached to the patient, as well as various other items commonly found in rooms such as gloves, and patient identifying information. For the ED scenario, the participant is told that the patient is awaiting a bed in the NICU. For the NICU scenario, the participant is told that the patient has just been admitted to the NICU and is getting settled. The room should be set up in disarray to mimic a post-earthquake environment with overturned items, drawers opened, phone off the hook, fake cracks in the walls, fake wall or ceiling tile on the floor, leaking water faucet, and other adjustments that you feel would add to the realism of the scenario. Once given instructions to prepare the patient for evacuation after an earthquake, the participant is asked to check themselves, the patient, and the environment for safety, repeat-back instructions for evacuation, and given five minutes to prepare the patient for evacuation. The scenario is completed once the participant begins to hand bag mask ventilate the patient, after five minutes have elapsed, or when the participant has stated that they are ready to evacuate. 

Scenario progression

Ideally, the scenario should start with the learner outside the room and receive a sign out on the patient. Afterward, as they enter the room, they are informed an earthquake has occurred and they find the room in disarray. The participant notes that they have been unharmed and state that the patient is apparently unharmed from the earthquake. The patient is intubated and remains hooked up to monitors and the ventilator with fluids and medications running. The room is upended with cracks on the wall, a leaky faucet, the telephone off the hook, the trash can overturned, the chair on its side, drawers/cabinets open, and debris on the floor. The participant notes that the room is unsafe. After being informed of the disaster code and need to evacuate the patient within five minutes, the participant repeats back the instructions utilizing a communication tool such as SBAR (Situation, Background, Assessment, Recommendation). The participant expediently prepares the patient for evacuation, and collects necessary items on the patient’s bed, collecting at least all critical items. Once ready to evacuate, the participant disconnects the patient from the ventilator and begins to hand bag mask ventilate the patient. The patient’s vital signs remain static during this scenario. The scenario concludes once the participant begins to hand bag mask ventilate the patient, when they indicate that they are ready to evacuate, or when five minutes have elapsed. If utilizing an embedded participant parent, the parent appears when the scenario has concluded and the participant will update the parent. The scenario progression is detailed in Table 3.

**Table 3:** Case changes and branch points

Case Changes and Branch Points	
Intervention / Time point	Additional Information
Prebriefing (outside the room-see history of present illness or HPI)	Manikin to be pre-set with the following vital signs: Heart Rate (HR) 160 SpO2 96% Blood Pressure (BP) 70/50 Respiratory Rate (RR) 44 Temperature (T) 37.5 degrees Celsius Ventilator settings: Assist Control (AC) 6 mL/kg; Positive End Expiratory Pressure (PEEP) 6, RR 30, Fraction of Inspired oxygen (FiO2) 0.45
Facilitator and participant enter the room and it is noted that an earthquake has occurred. Assess self, environment, and patient, utilize clear communication.	Facilitator: “I’m Liz, the charge nurse; there has been a major earthquake. Are you or your patient injured? Is there any damage to your room?” -Participant to visually assess self and patient and state that they are unharmed or okay. -Participant to state that there has been damage to the room (dependent upon what facilitator has set up the room to look like, for example, cracks in the wall, fallen ceiling tiles, spilled fluids, etc.)
Utilizing clear and safe communication tools such as Situation, Background, Assessment, Recommendation (SBAR) or repeat back closed loop communication (Appendix E).	Facilitator: “A disaster code is being activated and all communication should go through me as the code disaster Area Leader for our unit. Our unit has sustained major structural damage and we have multiple staff injured. We have the order to evacuate from the Emergency Operations Center to the hospital lobby where we will have shelter but minimal electricity and equipment due to damage to the building. You have five minutes to pack any necessary equipment and supplies for your patient. The pathway to the lobby is clear so you may move your patient on the bed. With some staff injured, we are spread very thin so you will need to work alone. When you are ready to go, disconnect from the ventilator and hand-bag your patient as we do not have staff to push the ventilators. Once you are packed and hand-bagging your patient, you may begin moving out. The scenario will end when you are ready to evacuate or when five minutes have passed. Please do a repeat back of my instructions.” -Participant to repeat back instructions in appropriate format. Example: Situation: My critically ill patient needs to be evacuated from this room which is now structurally unsafe. Background: There has been an earthquake and a code disaster has been activated. There are staff injured and so I will work alone to care for my patient. Assessment: My patient and myself are unharmed but the room is unsafe and so we must evacuate the room. Recommendation: I will prepare my patient for evacuation to the hospital lobby within five minutes. I will hand-bag the patient once the patient is ready to evacuate.
Collecting at least all essential items on evacuation checklist. Preparation to evacuation in less than or equal to five minutes. Effective hand bag mask ventilation of the patient as observed by facilitator when participant states they are ready to evacuate. *If utilizing an embedded participant parent, clear and open communication to the parent	After participant response above, say “Okay, your five minutes starts now,” start the timer and leave the room. The scenario ends after five minutes or when participant is ready to evacuate the patient. The critical items and actions checklists should be filled out and reviewed at the conclusion of the scenario along with the debrief. *If utilizing an embedded participant parent, may have the parent re-enter the room once the participant states that they are ready to leave and update the family -example update: “There has been a major earthquake, and Brandon has been unharmed in the disaster. However, the room is not structurally safe and so I have gathered essential equipment to evacuate him to the hospital lobby.”

This simulation is targeted toward one learner, and so the first step during or after the scenario is complete is to fill out the critical actions and items checklists. This is included with debriefing materials as we suggest that the facilitator do this with the participant so that the review can occur immediately post sim and can be concurrent with the debrief.

Facilitators may utilize the critical action checklist as below during the scenario to ensure that learners are meeting key action goals and critical items checklist (Figure 1) to ensure that essential items are being collected. These forms are particularly useful during participant debriefing to demonstrate to participants. 

Critical action checklist

❏ Visual safety assessment

❏ Participant assesses themself for safety

❏ Participant assesses patient for safety

❏ Participant assesses room for safety

❏ Repeat back

❏ Participant repeats back evacuation instructions utilizing a communication tool such as SBAR

❏ Evacuation

❏ Preparation in five minutes or less

❏ Critical Items (Essential items at least) -see next page for all items

❏ Face mask for bag

❏ Self-inflating bag

❏ Portable oxygen tank

❏ Code medication sheet

❏ Syringes with current medications running on pump

❏ Patient ID tag

❏ Pen or marker

❏ Communication device (e.g. cell phone or hospital mobile phone)

❏ Patient disconnected from ventilator and handbag masked

❏ If utilizing an embedded participant parent, the participant updates the parent at the conclusion of the scenario

Debriefing

Immediately after the learner has completed the simulation, debriefing occurs. Allow approximately 10 minutes for debriefing. The debriefing consists of reviewing the critical items and action checklists, as well as discussing communication and environment safety checking with the participant. The simulation session evaluation form (Appendix B) is used to obtain feedback on the simulation session from the participant. Supplemental educational material is optional, and the provided video slide-based lecture (Appendix C) and EVAC Checklist Card (Appendix D) can be used to help deliver supplemental content regarding evacuation of a critically ill neonate either before or after the simulation. Appendix D does not have to be used but may be helpful as a job aid for participants before, during, or after the simulation. Appendix E can be used as a reference for both facilitators and participants for common healthcare communication tools. 

## Discussion

This simulation curriculum is designed as a resource for nursing instructors to review evacuation of a critically ill neonate in the setting of an earthquake. This case is a high-risk, low-frequency scenario that is relevant to nurses caring for critically ill patients who may need to be evacuated in the case of a catastrophic event such as an earthquake. To be successful, learners must be familiar with the steps required at their institution to evacuate such a patient and adequately maintain that patient until help is available to assist.

This simulation case was used at our institution as part of a pediatric nursing disaster curriculum. We used this curriculum over eight days of training spread out over three months, in total with 60 pediatric nurses, 30 of whom were NICU nurses and 30 of whom were ED nurses. Overall, the curriculum received positive feedback from participants. After the first few iterations, based on timeliness and real-time participant feedback, we went over the evacuation items checklist with each participant immediately after the simulation. Most participants preferred going over the checklist together as opposed to filling out the checklist while the participant was filling out the simulation evaluation (Appendix B). While this was more time intensive, participants overall appreciated that they were able to debrief and go over the checklist simultaneously. One respondent suggested having a “cheat sheet” for evacuation, which we have subsequently created (Appendix D) that may be printed on a single page double-sided and downsized to ID card size. In addition, we were unable to capture every participant’s evaluation secondary to the time constraints of participants, although 58% of participants were able to fill out an evaluation. The curriculum received strong positive feedback via the evaluation forms. Learners agreed with statements “The simulation is relevant to my work,” “the facilitator created a safe learning environment,” “the simulation required critical thinking appropriate to my level of experience,” and “the debriefing was effective in identifying areas of improvement” (Table 4). The range of Likert Scores was three in “I was sufficiently oriented to the manikin and equipment before the simulation,” suggesting that some participants felt inadequately oriented to the simulation equipment. In our scenario, it was difficult to orient participants to the manikin prior to the simulation as orienting a participant would have introduced them to the room earlier than the scenario allowed and thus may have affected the simulated evacuation process. If a facility has the resources to have an extra manikin, it might be useful to orient participants to a manikin outside of the simulation room. All but one participant felt that they learned something from the simulation, but that respondent did not specify further in comments. In particular, learners felt that this scenario was useful in prioritizing items for patient evacuation and in differentiating what was essential. Furthermore, learners remarked that they felt better prepared to safely evacuate a critically ill neonate in the setting of a disaster in terms of closed-loop communication, equipment prioritization, patient/self-safety, and efficiency.

**Table 4:** Participant evaluations 1 = strongly disagree, 2 = disagree, 3 = neutral, 4 = agree, 5 = strongly agree *N = 34

	Mean Likert Score (N = 35)	Median Likert Score (N = 35)	Range of Likert Scores (N = 35)
This simulation is relevant to my work.	4.77	5	4-5
I was sufficiently oriented to the manikin and equipment before the simulation.	4.26	4	2-5
The facilitator created a safe learning environment.	4.83	5	4-5
The simulation required critical thinking appropriate to my level of experience.	4.60	5	4-5
The facilitator was effective in teaching skills appropriate to my level of experience.*	4.68	5	4-5
The facilitator was effective in teaching teamwork and communication skills.	4.43	5	3-5
The debriefing was effective in identifying areas of improvement.	4.49	5	3-5

Adjustments were made based upon the environment being simulated. For instance, it was useful to add an arterial line to the patient if the setting was the NICU in order to maintain realism, whereas in the ED setting at our institution, arterial lines are rare and including one with an arterial line in the patient was not useful in the ED scenario. This scenario is designed for individual learners to mimic the realism of evacuating their patient alone. Moving forward, it might be worthwhile to have an individual learner prepare two patients for evacuations, as this might occur in an earthquake scenario. A limitation we found was time, as the scenario is designed for one individual, thus it is a significant time investment to run through the curriculum with all of the nurses consecutively. However, we felt that the simulation needed to be designed for one individual in order to maintain realism. In addition, all of the simulations were run in our simulation center (versus in situ in the NICU or the ED), which can make it difficult to suspend disbelief. In spite of these negatives, this scenario is now part of the pediatric disaster simulation series curriculum at our institution as our insitution's leadership is invested in high-yield disaster education as it is located in an earthquake prone zone. We are currently working to create a less time and resource-intensive scenario that may be completed asynchronously such as through virtual simulation to make it more accessible for our nursing workforce. While in-situ simulation is ideal, it is not always feasible secondary to institutional constraints. This scenario did not specifically go over communication with parents, so we suggest that adding in a parent as an embedded participant or asking the participant at the end of the scenario to state how they would communicate with the parent. Table 5 reviews anticipated management mistakes that our learners encountered and suggested ways to mediate them. Additionally, Appendix A contains debriefing materials and techniques.

**Table 5:** Anticipated management mistakes SBAR = situation, background, assessment, recommendations

Anticipated Management Mistakes	
Failure to assess the situation	After being informed of the earthquake, most learners responded with “no” to “Are you or your patient injured?” and “Is there any damage to your room?” without actually looking at the environment. We found that reviewing the concept of visually assessing yourself, the patient, and the room during the debrief to be helpful. Prompting the participant with the qualifier, “When you look around at your environment…” may also be helpful.
Difficulty using safe communication tools	While most participants were able to communicate their status, not everyone used commonly accepted communication tools such as SBAR. We found it helpful to review communication tools (Appendix E) before the simulation or during the debrief.
Failure to collect appropriate items for transport of critically ill patient	Most participants collected critical items. It may be helpful to review bedside items for transport of a critically ill neonate prior to the simulation. We incorporated this into our debrief. If a goal is to incorporate the checklist as something that learners carry with them in real life scenarios, we suggest that learners be handed a checklist prior to the simulation (Appendix D).
Difficulty hand bag mask ventilating	A majority of our learners adequately disconnected the patient from the ventilator and hand bag mask ventilated. It may be useful to review this prior to the simulation if learners do not have experience with this task.

This simulation is resource-intensive, as it requires extensive environmental preparation in order to meet curricular goals. It may be adjusted for use with less environmental staging for the earthquake, although these changes may compromise the realism of the case. This could be expanded to be a group simulation using a larger number of rooms and manikins to simulate evacuating a full unit.

## Conclusions

The implementation of a low-frequency high-impact disaster-related simulation to efficiently evacuate a critically ill neonate and pack critical equipment was well received by bedside nurse learners from the NICU and PED. Implementing the scenario allowed nurse participants to physically practice packing critical equipment, utilize resource management, and practice standardized communication skills. While this scenario may not work for every institution, it can be performed with varying degrees of fidelity and can be expanded to a group simulation model, as well as incorporated as a hands-on aspect of pediatric disaster nursing curriculum, which is typically lecture-based.

## Figures and Tables

**Video 1:** EVAC simulation slides-based didactic

**Figure 2:**
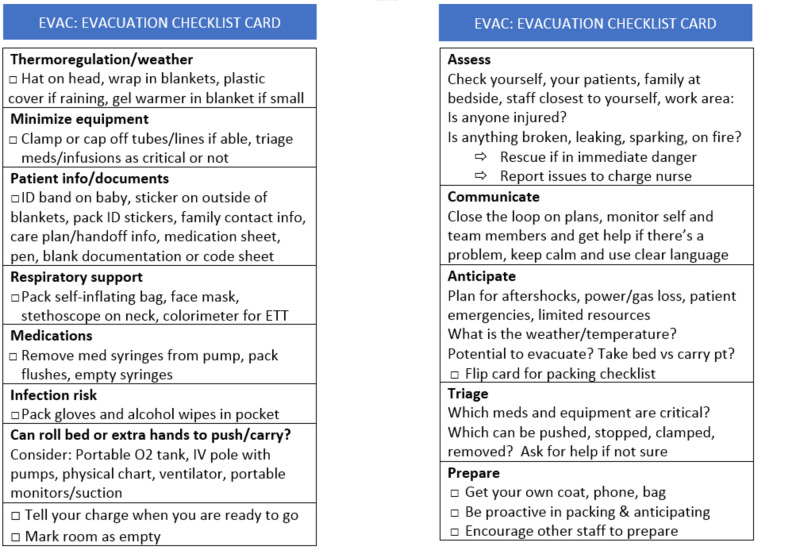
EVAC participant checklist card This table can be formatted into card format to hand out to participants during the debrief. It can be shrunk and folded to fit in an ID card holder.

## Appendices

Appendix A: EVAC simulation debriefing materials

This appendix contains a debriefing overview for facilitators to utilize during the simulation and debrief. 

Debriefing Overview

This simulation’s debrief allows the learner to go over medical management, hands-on skills, and communication in order to improve patient care.

Debriefing Outline: We suggest that the facilitator goes over the critical action and items checklists immediately after the simulation has completed (see Technical Report).

Sample script:

Facilitator: “This checklist of bedside items was created in conjunction with experienced pediatric nurses. The essential items should be packed for all critically ill patients, while the non-essential items may delay time to evacuation.” It looks like you collected __ number of essential items, __ number of fairly important items, __ number of less important items, and ___ number of non-essential items.”

Facilitator may subsequently review which essential items were missed. If all items were collected, you may prompt, “What helped you remember to collect all of these items?” If essential items were missed, the facilitator may prompt, “What hindered you from collecting this item?”

Appendix B: EVAC simulation session evaluation form

This evaluation form can be given to participants after debriefing to allow for feedback. 

Instructor: 

Date: 

**Table 6:** EVAC: Simulated nurse-led evacuation of critical and vulnerable pediatric patients evaluation

	Strongly Disagree	Disagree	Neutral	Agree	Strongly Agree
This simulation is relevant to my work.	1	2	3	4	5
I was sufficiently oriented to the mannequin and equipment before the simulation.	1	2	3	4	5
The facilitator created a safe learning environment.	1	2	3	4	5
The simulation required critical thinking appropriate to my level of experience.	1	2	3	4	5
The facilitator was effective in teaching skills appropriate to my level of experience.	1	2	3	4	5
The facilitator was effective in teaching teamwork and communication skills.	1	2	3	4	5
The debriefing was effective in identifying areas of improvement.	1	2	3	4	5

What did you learn that will change your future practice?

What could have made this simulation more effective?

Additional comments: 

Appendix C: EVAC simulation slides-based didactic

This video may be used to supplement learning either prior to or after the simulation. 

Appendix D: EVAC simulation participant checklist card

Appendix E: Pediatric disaster simulation communication tools

Common communication terminology used in healthcare and medical simulation settings. 

**Table 7:** Common healthcare communication phrases Adapted from: Bartman T, McCLead RE. Core Principles of Quality Improvement and Patient Safety. Pediatrics in Review Oct 2016, 37 (10) 407-417; DOI: 10.1542/pir.2015-0091 U.S. Department of Health and Human Services (HHS) Agency for Healthcare Research and Quality (AHQR) and Department of Defense (DoD) Team Strategies & Tools to Enhance Performance & Patient Safety (TeamSTEPPS): TeamSTEPPS 2.0. Content last reviewed August 2018. Agency for Healthcare Research and Quality, Rockville, MD. http://www.ahrq.gov/teamstepps/instructor/index.html Weick KE, Sutcliffe KM. Managing the Unexpected: Resilient Performance in an Age of Uncertainty. San Francisco, CA: Jossey-Bass; 2007

Term	Definition
Check-back or closed-loop communication	Verification of communicated information. A message is initiated, then restated by the intended recipient, and that restatement is acknowledged/verified by the sender. (Example: “Give a 20 mL/kg normal saline bolus IV push” – “20 mL/kg normal saline mg IV push” – “That’s correct”)
CUS	Utilizing the key phrases “I am Concerned, I am Uncomfortable, This is a Safety Issue” in order to clearly understand the gravity of the issue raised.
Debrief	An informational/educational session designed to improve participant performance in the scenario.
Pre-Brief	A brief discussion prior to the start of the scenario to assign roles, establish expectations and expected outcomes.
QVV	When unsure of next steps, qualify the source, validate the source, and verify understanding.
SBAR	An outline of succinct communication. S = Situation (What is the patient’s situation?) B = Background (What is the context?) A = Assessment (What is the issue?) R = Recommendation (What would I recommend to correct it?)
